# Perceived Religious Value Similarity and Mother-Adult Child Relations: Exploring Variations by Generation

**DOI:** 10.1093/geroni/igaf122.953

**Published:** 2025-12-31

**Authors:** Destiny Ogle, J Jill Suitor, Ranran He, Robert Frase, Megan Gilligan

**Affiliations:** Purdue University, West Lafayette, Indiana, United States; Purdue University, West Lafayette, Indiana, United States; Purdue University, West Lafayette, Indiana, United States; Southern Illinois University, Carbondale, Illinois, United States; University of Missouri, Columbia, Missouri, United States

## Abstract

Research on interpersonal relations has emphasized the importance of value similarity in the formation and maintenance of ties across the life course. Consistent with this body of theoretical and empirical work, religious similarity has been highlighted as a predictor of the quality of relationships between parents and adult children. However, most studies have conceptualized religious similarity in terms of denominational congruence, rather than perceptions of religious similarity. Moreover, despite evidence that religion is less salient for younger generations, no consideration has been given to the role that generation plays in the relationship between perceptions of religious value similarity and parent-child relationship quality. In this paper, we explore the association between perceptions of religious value similarity and mother-child closeness and tension using data collected from adult children in two generations of the same 147 families as part of the Within-Family Differences Study-III. Specifically, we compare the associations between perceptions of religious value similarity and relationship quality in ties between older mothers (Mage=88) and their midlife adult children (Mage=59), and midlife mothers (Mage=59) and their young adult offspring (Mage=29). Regression analyses revealed that adult children’s perceptions of religious value similarity were associated with mother-child closeness and tension in both generations. However, these associations were stronger for ties between midlife children and their mothers than for ties between younger adult children and their mothers. These findings highlight the importance of religious value similarity for the quality of mother-adult child ties across generations and the greater impact of such similarity in later-life families.

